# Probing nanoscale damage gradients in ion-irradiated metals using spherical nanoindentation

**DOI:** 10.1038/s41598-017-12071-6

**Published:** 2017-09-20

**Authors:** Siddhartha Pathak, Surya R. Kalidindi, Jordan S. Weaver, Yongqiang Wang, Russell P. Doerner, Nathan A. Mara

**Affiliations:** 1 Chemical and Materials Engineering, University of Nevada, Reno, NV 89557 USA; 2 George W. Woodruff School of Mechanical Engineering, Georgia Institute of Technology, Atlanta, Georgia, GA 30332 USA; 3 Center for Integrated Nanotechnologies, Los Alamos National Laboratory, Los Alamos, NM 87545 USA; 4 Materials Science and Technology Division, Los Alamos National Laboratory, Los Alamos, NM 87545 USA; 5 Center for Energy Research, University of California at San Diego, La Jolla, CA 92093 USA; 6 Institute for Materials Science, Los Alamos National Laboratory, Los Alamos, NM 87545 USA

## Abstract

We discuss and demonstrate the application of recently developed spherical nanoindentation stress-strain protocols in characterizing the mechanical behavior of tungsten polycrystalline samples with ion-irradiated surfaces. It is demonstrated that a simple variation of the indenter size (radius) can provide valuable insights into heterogeneous characteristics of the radiation-induced-damage zone. We have also studied the effect of irradiation for the different grain orientations in the same sample.

## Introduction

Materials with modified surfaces – either as a consequence of a graded microstructure or due to an intentional alteration of the surface such that its physical, chemical or biological characteristics are different from the bulk of the material – are of increasing interest for a variety of applications such as enhanced wear and corrosion resistance, superior thermal and biomedical properties, and higher fracture toughness^1,2^. In some cases such gradations at the surface may also be caused unintentionally as a consequence of the service life of the material, such as in wear applications^3^ or irradiated materials which show varying degrees of radiation damage that change with depth, location of radiation source, etc.^4^. Quantifying the resulting property gradations poses a significant challenge, especially when the changes occur over small (sub-micrometer) depths. In this communication, we present a novel indentation approach, which together with the corresponding local structure information obtained from electron back-scattered diffraction (EBSD), allows us to probe nanoscale surface modifications in solid materials and quantify the resulting changes in its mechanical response.

The study of mechanical degradation in the surface layers of ion-irradiated materials is an example of one such outstanding challenge for which few practically viable solutions^4–7^ exist. In materials undergoing irradiation in reactor or spacecraft applications, the resulting damage is often highly heterogeneous (with strong gradients normal to the surface) depending on component location as well as the nature of the irradiation source itself. In nuclear materials research, reactor conditions can be mimicked using ion beams where large amounts of radiation damage (several displacements per atom (dpa)) are imparted in relatively short time spans of hours or days that would require months or years to achieve in reactor conditions^8–10^. However, the volume of ion-irradiated material is limited by the beam energy to depths of fractions of a micron to several microns, making the investigation of bulk mechanical properties very difficult. A key challenge then becomes: “How can we study the mechanical response of materials with varying degrees of damage over scales of only a few hundreds of nanometers in such a way that the data can be related to bulk values?” The very small thickness of irradiated material, high level of damage heterogeneity, sensitivity to sample preparation techniques, and the time and effort needed for sample preparation and testing, often preclude the application of many of the commonly used nano-mechanical test techniques; these include the use of focused ion beams (FIB) to fabricate micro-pillars or other small scale test geometries^5–7,11–16^.

Among the experimental techniques available at these length scales, nanoindentation, with its high resolution load and depth sensing capabilities, shows the greatest promise due to its non-destructive nature, ease of experimentation (only a polished surface prior to ion irradiation is needed) and versatility^4,5,17–19^. In particular, using spherical indenters, our recent work^20–22^ has demonstrated the feasibility of transforming the raw load-displacement data into meaningful indentation stress-strain curves (see Eqs 1 and 2 in the ‘Materials and Methods’ section). These indentation data analysis methods have captured successfully the local loading and unloading elastic moduli, the local indentation yield strengths, and certain aspects of post-yield strain hardening behavior in various polycrystalline metal samples^23^. More specifically, the use of these indentation stress-strain curves makes it possible to analyze the initial loading segments of spherical indentation – before the indentation itself imposes additional local plastic deformation and alters the local microstructure and its properties. Coupling the mechanical data obtained from nanoindentation with the structure information obtained from EBSD has also provided new insights into the local elastic-plastic properties of interest^22,24,25^. This has enabled the measurement of the local indentation yield strengths in individual grains of deformed polycrystalline metallic samples^26–28^, and across their grain boundaries^29^, which in turn can be related to percentage increases in the local slip resistances from their fully annealed conditions. Recent reports have also used these ﻿﻿and other ﻿related﻿ techniques for studying irradiated nuclear materials^15,30,31^. In this communication, we apply these methods to indentations on ion-irradiated metallic materials, and compare their relative mechanical behavior to the unirradiated state.

**Figure 1:**
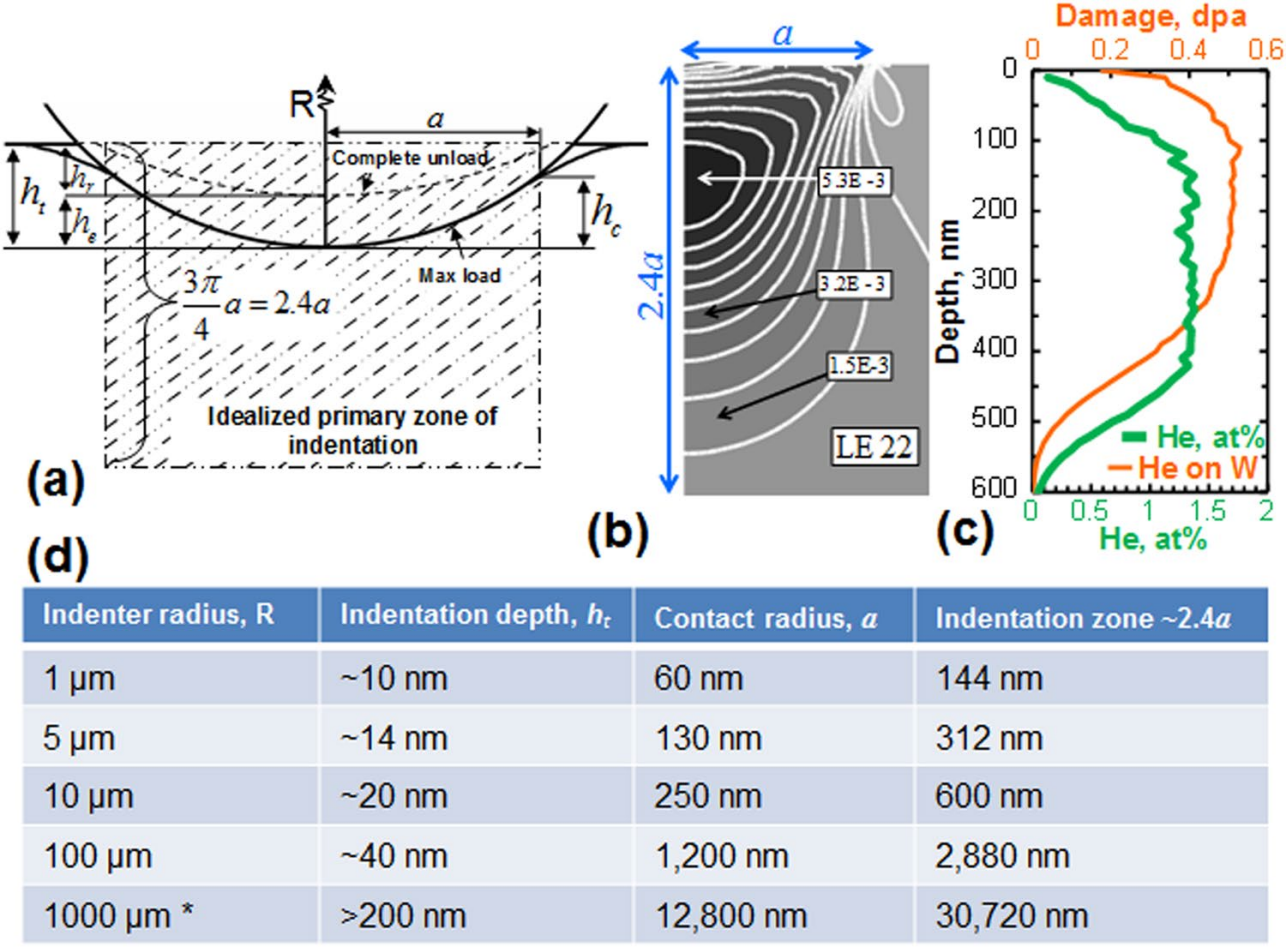
(**a**) Schematic of spherical indentation showing the idealized primary zone of indentation. (**b**) Logarithmic strain field (along the indentation direction) for a spherical indenter in the indentation zone (~2.4*a*, where *a* is the contact radius) close to the indentation yield. (Reprinted from^40^, with permission from Elsevier.) Both the contact radius *a*, and hence the volume probed by indentation, can be controlled with a proper choice of indenter radii. This approach is thus ideally suited for measuring any mechanical changes in the modified material surface layers, such as probing the (**c**) damage caused by He irradiation on a tungsten sample. (**d**) Table showing indentation depth (*h*_*t*_), contact radius (***a***) and indentation zone size (~2.4*a*) at yield for W using 5 different indenter radii. *For the 1000 µm radius indenter, the response was all elastic up to h~200 nm (instrument limit).

**Figure 2:**
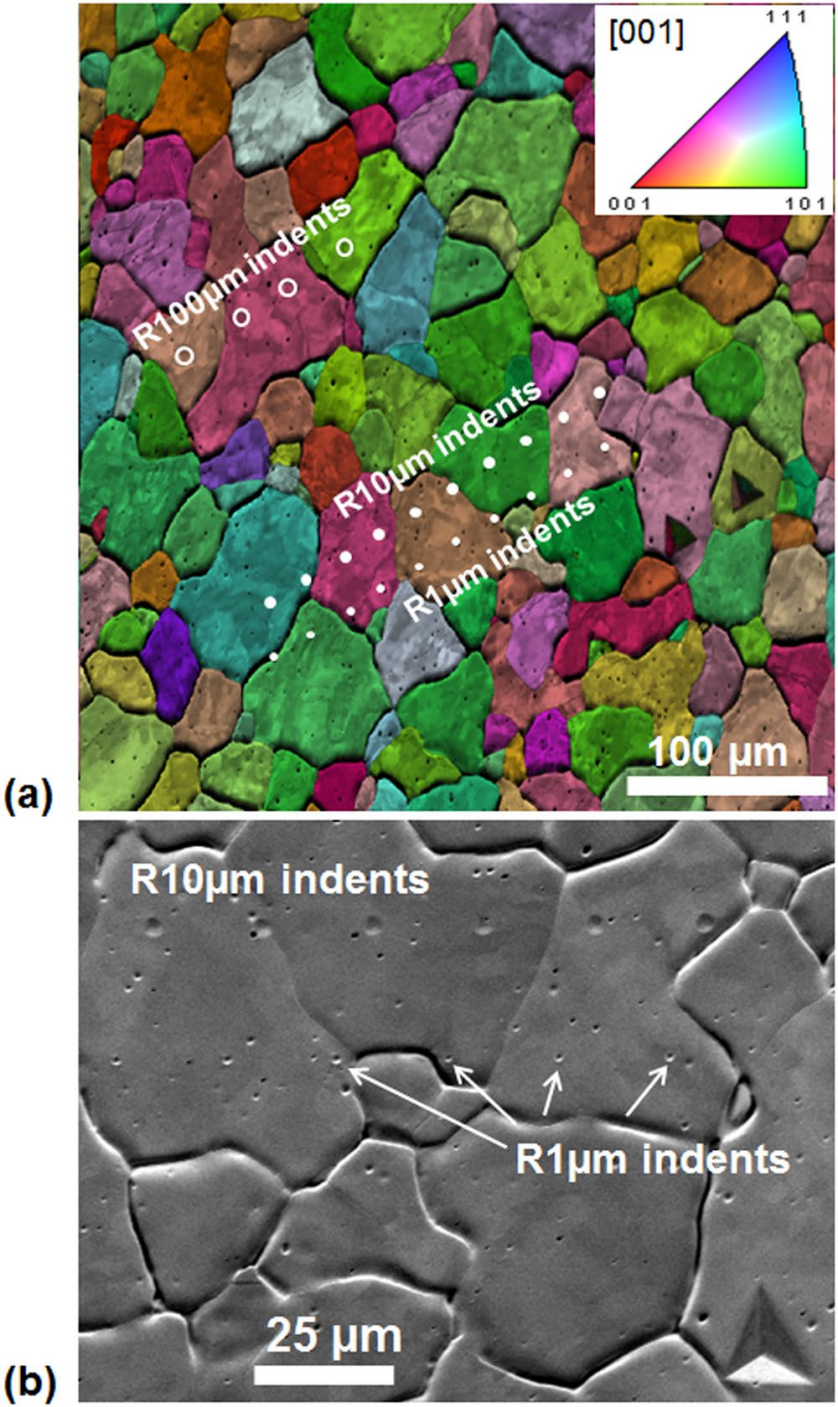
(**a**) EBSD image quality map with superimposed grain coloring using the inverse pole figure (IPF, shown in inset) scheme, showing the indent locations for different indenter sizes of 1, 10 and 100 µm radii. (**b**) A magnified SEM image showing the indent locations for the 1 and 10 µm radii indenters.

The use of spherical indenters also presents an important opportunity to systematically study responses at different material volumetric or length scales – by simply varying the indenter radii. This concept has been explored by various research groups including ourselves^22,32^ and teams from Drexel University^33,34^, Oak Ridge National Laboratory^35–37^ and others^31,38,39^, and is briefly described below. Since Eqs 1 and 2 analyze the initial loading segments of the indentation datasets, we can systematically vary the indentation zone sizes at yield (i.e., at the point where the indentation zone is dominated by plastic yielding) in the range of 100 nm to >30 μm by using a range of indenter tip radii. This is depicted in the table in Fig. 1d, which shows the approximate indentation depth (*h*_*t*_) and the corresponding contact radius (*a*) and the depth of the indentation zone (which scales as 2.4*a*, see Fig. 1a,b ^20^) at yield in annealed tungsten for five different indenter radii. This table illustrates the need for a proper choice of the indenter size in order to closely correspond the volume probed by nanoindentation (Fig. 1a,b ^40^) to the depth of He radiation-damaged region (Fig. 1c). The use of four different sized indenter radii (*R*_*i*_ = 1, 5, 10, and 100 µm, see Fig. 2) can also reveal some of the salient features of the inherent heterogeneity expected in the sample (in the depth direction). Furthermore, the ability to make a large number of measurements on a given sample surface also has the potential to provide quantitative information on the variance of properties in the irradiated layer.

**Figure 3:**
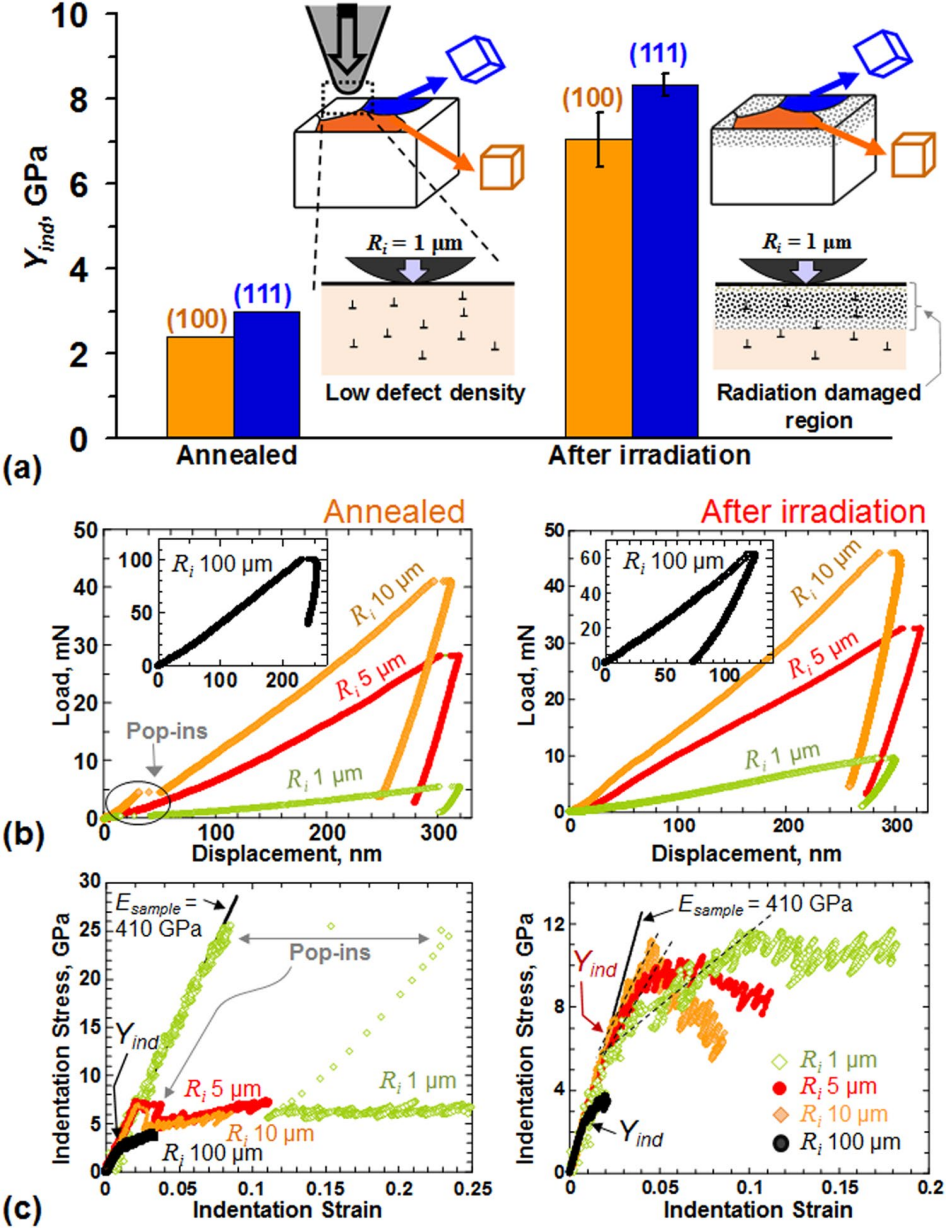
(**a**) Causes for the change in *Y*_*ind*_. In annealed electro-polished tungsten, the defect density is low across all grains. Here *Y*_*ind*_ varies from one grain to another mainly due to the differences in the activities of the different slip systems in the different grains and their orientation with the indentation direction. Upon ion-irradiation, the metal surface is modified by a damaged layer, which causes a change in its mechanical response as compared to the bulk of the sample. The *Y*_*ind*_ in irradiated samples therefore depends on both the grain orientation and the interaction of the indentation zone with the radiation damaged layer at the indentation site. Typical (**b**) load-displacement and (**c**) indentation stress-strain responses for a near (001) grain in annealed electro-polished tungsten using 4 different indenter sizes of radii 1, 5, 10 and 100 µm before and after He irradiation.

Figure 3 demonstrates the capability of spherical nanoindentation in reliably characterizing the grain-scale heterogeneities present in metallic materials. Since the length scales in our spherical nanoindentations are much smaller than the typical grain sizes of 10 to 60 µm (average grain size 35 µm) in our tungsten sample, the local lattice orientation(s) at the indentation site (measured using EBSD) are expected to strongly influence the elastic-plastic properties of the indents (see Fig. 2)^26,41,42^. These differences arise because of the inherent differences in the local material structure at the indentation site. For example, although tungsten is elastically isotropic, it is fully expected that the indentation yield strength (*Y*_*ind*_) in tungsten will vary significantly from one crystal orientation to another, even in fully annealed samples where there are no major differences in the dislocation content of the differently oriented grains. This is because the local plastic deformation imposed by the indenter needs to be accommodated locally at the indentation site by slip activity on the available slip systems, whose orientation and activation are strongly dependent on the local crystal lattice orientation with respect to the indentation direction (see the illustration in Fig. 3a).

Additionally, upon ion-irradiation the metal surface is modified by a thin radiation-damaged layer (see Fig. 3a), which causes a change in its mechanical response as compared to the bulk of the sample. The *Y*_*ind*_ in irradiated samples therefore depends on both the grain orientation and the extent of radiation damage at the indentation site. In order to successfully study the effects of radiation damage on the indentation behavior, we need to first decouple the effects of orientation from the effects of the increased defect density caused by irradiation.

Figures 3b,c show the comparison of the indentation load-displacement and indentation stress-strain responses respectively for grains whose surface normals were very close (within 6 degrees) to [100] directions. These grains were purposely selected to avoid the need to correct for the effect of the lattice orientation at the indentation site in comparing the different measurements presented in these plots. Of particular interest in the tests on the annealed sample is the occurrence of ‘pop-in’ events, which are seen as sudden excursions in indentation depth in Fig. 3b and as indentation strain bursts in Fig. 3c. Commonly referred to as indentation size effect (ISE), pop-ins are known to act as a trigger for the onset of plastic deformation^36–38^, with the stresses under the indenter reaching extremely high values (approaching the theoretical shear strength of the material) before the pop-in event. Their cause has been generally attributed to the difficulty of activating potent dislocation sources (e.g., Frank-Read sources)^37,43^ in the very small indentation zones (typically much smaller than the length scales associated with dislocation spacing or dislocation cell size) in these experiments^44^. This physical explanation is consistent with the observations that the pop-ins occur most readily in indentation experiments on annealed samples which have very low defect density^45,46^. Their propensity should decrease with increasing indentation zone size^13,22,36,47^ – which increases the likelihood of encountering dislocation sources within the indentation zone. Consistent with this assertion, the very small indenter tip radii of *R*_*i*_ = 1 µm shows the largest indentation strain burst in Fig. 3c, with the burst size decreasing for larger indenter sizes of *R*_*i*_ = 5 µm and *R*_*i*_ = 10 µm, while the pop-in completely disappears for the largest *R*_*i*_ = 100 µm indenter size^48,49^. Similar behaviors have also been reported in literature, where large pop-ins are observed for the smaller indenter radii (R = 0.38 and 3.8 µm), with the pop-in size decreasing for larger indenters^50^. Thus, unlike sharp pyramidal indenters (e.g. Berkovich, cube corner etc.)^51^, the ISE for spherical indenters is manifested not through the depth of penetration but rather through the radius of the sphere^52^.

Pop-ins in annealed materials can sometimes be avoided by choosing a different final polishing step, such as vibratory polishing (instead of electro-polishing) in tungsten, which has been shown to eliminate pop-ins without significantly affecting the *Y*_*ind*_^29,46^. If pop-ins are present, back-extrapolation of the post-pop-in portion of the indentation stress-strain curves for these indenter sizes should also generate a similar *Y*_*ind*_ value as measured with the larger *R*_*i*_ = 100 µm (that does not have pop-ins). However back-extrapolation can sometimes be tricky, especially if the pop-in causes too large a discontinuity in the indentation stress-strain curve (as in Fig. 3c for *R*_*i*_ = 1 µm). This makes it difficult to accurately estimate *Y*_*ind*_ from such a plot. It then becomes necessary to use a large indenter radii such as *R*_*i*_ = 100 µm, where pop-ins are absent, to reliably measure *Y*_*ind*_ in annealed electro-polished metals.

We note that Fig. 3c clearly shows that for annealed W, the indentation stress-strain curves overlap for the 4 different indenter sizes (*R*_*i*_ = 1, 5, 10, and 100 µm) shown, if one were to ignore the initial pop-in for the smaller indenter sizes. In other words, once the indenter has penetrated past the initial pop-in, there is no further evidence of ISE in these tests on annealed tungsten. Similar trends showing a consistent indentation stress-strain response for indenters of different sizes have also been reported by others^31^. However multiple reports in literature have also noted a different trend, where the indentation stress appears to increase as the indenter radius decreases^50,52,53^. These differences can be attributed to the different indentation analysis protocols that were followed in these reports as compared to Eqs 1 and 2 of this manuscript. Our recent published works^22,40^ provide a more comprehensive discussion on these topic.

The indentation stress-strain curves from the irradiated samples (Fig. 3c) also reveal several novel features. Strikingly, none of the measurements in the irradiated sample (including the measurement with the smallest indenter tip *R*_*i*_ = 1 µm) revealed any pop-ins. A possible explanation could be that the ion-irradiation introduces a large density of defects (such as dislocation loops^54^, or He bubbles, etc.) into the material structure that can help set up highly potent dislocation sources, as reported by numerous indentation studies on ion-irradiated materials^15,55–57^. Another obvious consequence of these new defects introduced by irradiation is that the *Y*_*ind*_ values in the irradiated samples are higher than the corresponding values in the annealed samples. It is also observed that in addition to the higher yield values, the irradiated samples are exhibiting more complex features (e.g., multiple distinct regimes of hardening/softening) compared to the annealed samples.

**Figure 4:**
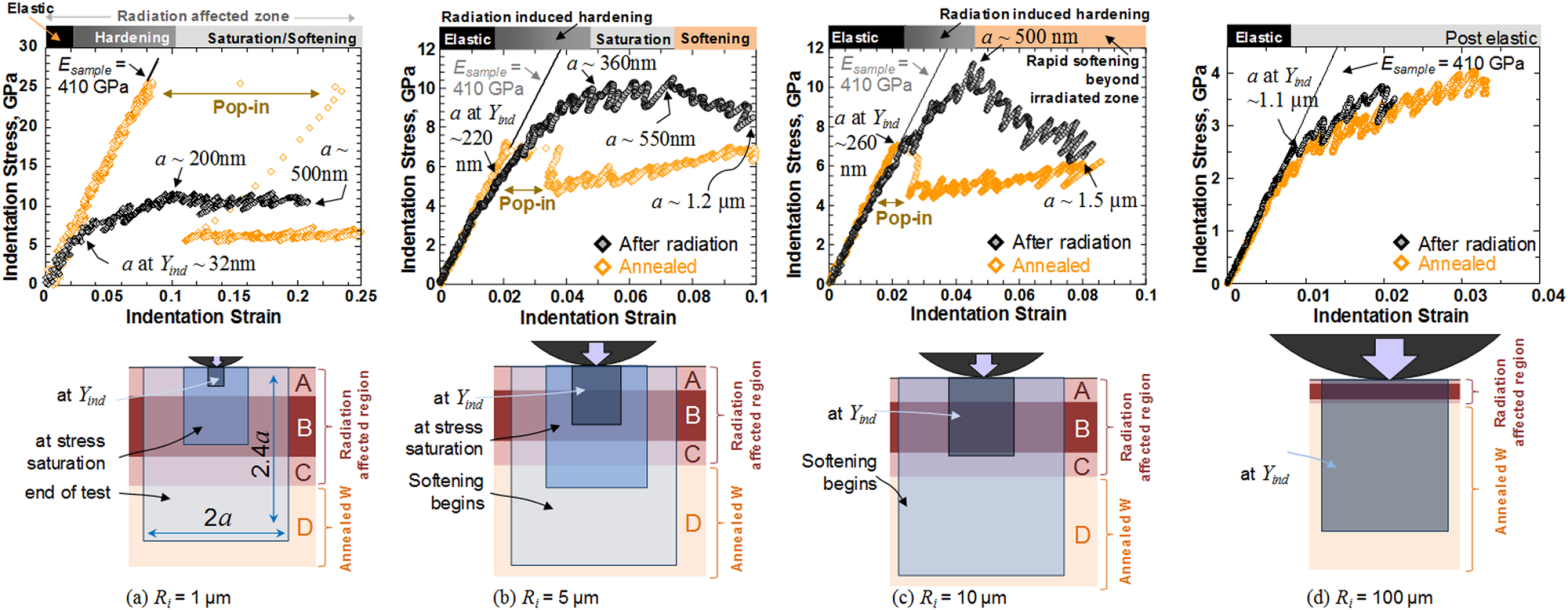
Comparing the indentation stress-strain responses between annealed (orange curve) and irradiated (black curve) W grains of near (001) orientation for four different indenter tip radii (**a**) 1 µm, (**b**) 5 µm, (**c**) 10 µm and (**d**) 100 µm.

**Figure 5:**
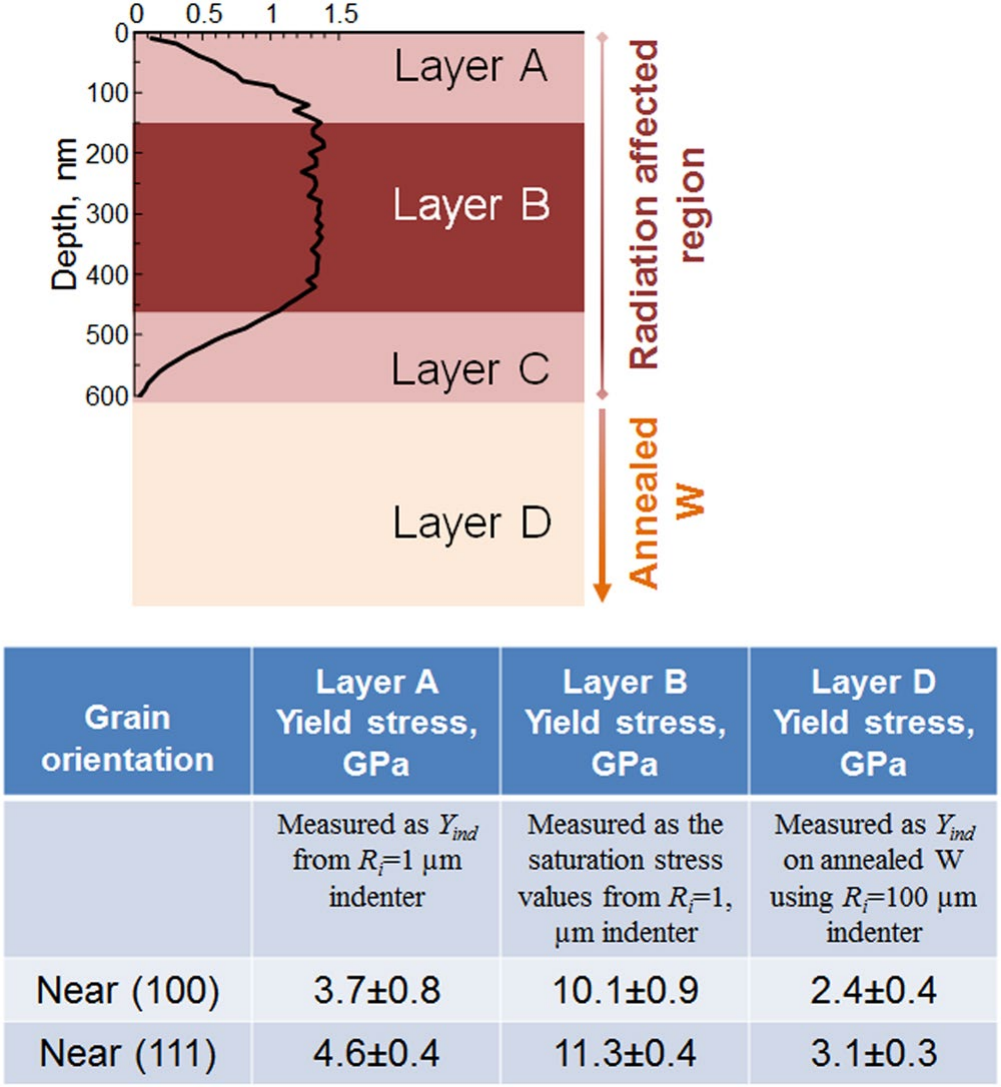
Schematic showing the He radiation affected region idealized into four layers labelled (**A**) through (**D**). The bottommost layer (**D**) denotes the virgin (undamaged and annealed) material below the radiation affected region. The table summarizes the results for the indentation measurements performed on the annealed and He irradiated tungsten samples.

The differences in indentation stress-strain response before and after irradiation are examined for each indenter size in Fig. 4a–d. Each figure also provides a schematic depiction of the estimated evolving indentation volume at different points of the test^22,40,58^. Here the primary zone of indentation deformation is idealized as a cylindrical region of radius *a* and height 2.4*a*, where *a* is the indentation contact radius, with the highest indentation stresses being expected between depths of ~*a*/2^40,58,59^ to *a* below the indenter (see schematic in Fig. 4a–d). The evolution of *a*, and by extension that of the primary indentation zone size, is related to the increase in indentation depth and load according to Eq. 2. Additionally, the radiation-damaged region is idealized into four layers labelled A through D (see table in Fig. 5) delineating the different zones of radiation damage based on the profile of the imposed He damage concentration. Layer B denotes the region between 150 nm and ~450 nm, where the He concentration in W was estimated to be the highest (~0.92 atomic%). Layers A and C indicate transition regions with strong damage gradients. The bottommost layer D denotes the virgin (undamaged and annealed) material below the radiation affected region.

Figure 4a indicates that the initial indentation yield in the test conducted with the *R*_*i*_ = 1 µm indenter tip occurs at a contact radius of *a* ~ 32 nm. Thus the indentation zone at yield for this test comprises largely of the transition layer A, and the measured *Y*_*ind*_ value provides an estimate for the effective indentation yield strength for layer A as 3.7 ± 0.8 and 4.6 ± 0.4 GPa for near-(100) and near-(111) oriented grains, respectively (see table in Fig. 5). With continued loading, the indentation volume increases and gradually includes layer B, which has experienced the highest level of radiation damage. This results in apparent hardening in the indentation stress-strain curve in Fig. 4a between indentation strains of 0.03 and 0.1, i.e., the indentation flow strength appears to increase with indentation strain. It is interesting to note that at about an indentation strain of 0.1 (where *a* ~ 200 nm), the indentation flow strength reaches a saturation value of around 10.5 GPa. There is no further strain hardening detected beyond this point, although the indentation zone is now primarily comprised of the heavily damaged layer B. There is a slight softening in the flow strength towards the end of the test, where the indentation zone now extends to include layers C and D, both of which are expected to be softer than layer B.

The saturation value of indentation flow strength from this test reflects largely the strength of layer B. In other words, if layer A were absent, *Y*_*ind*_ from this indentation test would be close to the saturation flow strength value of 10.1 ± 0.9 GPa (average ± standard deviation for near-(100) oriented grains; see table in Fig. 5). Note that this value is 3.2 times higher than the *Y*_*ind*_ of annealed W grains of similar orientation. Large increases in plastic flow strengths are generally accompanied with loss of strain hardening and formation of instabilities such as shear banding^60–62^. We therefore hypothesize that layer B exhibits a high indentation plastic flow strength, but very little real strain hardening under the indenter. There is clearly a need to conduct detailed microscopy studies (most likely transmission electron microscopy (TEM) studies) to confirm this hypothesis. It is indeed remarkable that the indentation stress-strain protocols employed in this work are capable of providing meaningful insights into the local mechanical response of the damaged layers in the sample and guide future efforts.

Figure 4b, corresponding to indentation with the larger *R*_*i*_ = 5 µm indenter tip further confirms the observations outlined above. Here the indentation zone sizes are significantly bigger compared to the test with the *R*_*i*_ = 1 µm indenter tip (compare Fig. 4a and b), and hence different regimes are substantially compressed. For example, the contact radius at yield (*a* ~ 220 nm) is substantially larger, and hence the indentation zone at yield encompasses both layers A and B. The *Y*_*ind*_ value of 6.1 ± 0.8 GPa for near-(100) oriented grains (and 6.9 ± 0.4 GPa for near-(111) oriented grains) from the *R*_*i*_ = 5 µm tests thus reflect contributions from both these layers. Also, the regime of initial apparent hardening is now significantly shorter and the saturation level of the indentation flow strength is reached earlier (at about an indentation strain of 0.04). It is indeed significant that the saturation value of the indentation flow strength for this larger *R*_*i*_ = 5 µm indenter is around 9.9 ± 0.9 GPa for near-(100) oriented grains and 9.4 ± 0.5 GPa for near-(111) oriented grains; highly consistent with the earlier observations from the tests with the *R*_*i*_ = 1 µm indenter tip (Fig. 4a). This good agreement between the values of the saturation flow strengths from the two different indenter sizes supports our earlier hypothesis of a high yield strength for layer B without significant hardening. Finally, it is also reassuring that the indentation flow strength in this test at large indentation strains shows significant apparent softening and actually approaches the indentation stress-strain curve measured from the annealed sample. Indeed, the contact radius towards the end of the test is about 1.2 µm (see Fig. 4b). Therefore, at this high indentation strain level, the indentation zone now extends significantly into the annealed layer D comprising of the softer material below. It is interesting to note that the transition between the saturation and softening regimes in this indentation stress-strain curve occurs at a contact radius of ~550 nm, which is comparable both to the depth of layer C in the radiation affected region (see Fig. 5), and TEM investigations of the depth of He bubble depth (~500 nm).

Increasing the indenter size to *R*_*i*_ = 10 µm further confirms the trends described above (Fig. 4c). The initial indentation yield is now higher (*Y*_*ind*_ = 6.4 ± 0.5 GPa and 7.4 ± 0.8 GPa for near-(100) and (111) oriented grains respectively), the apparent indentation strain hardening and saturation regimes are shorter, and the subsequent softening regime is even more dominant. Interestingly, the peak indentation flow strength is still around 10 GPa (9.9 ± 0.8 GPa and 9.2 ± 0.5 GPa for the near (100) and near (111) grains respectively), lending support to our earlier hypotheses regarding the mechanical response of layer B. The contact radius at transition between the hardening and softening regimes of the indentation stress-strain response (*a* ~ 500 nm) also matches the depth of the radiation-affected region. As expected, the indentation stress-strain curves from the annealed and the irradiated samples show excellent convergence at larger indentation strains. All of the observations described are a testament to the unique potential of the indentation stress-strain protocols in obtaining meaningful insights into the local mechanical response from exceedingly small volumes.

Increasing the indenter size further to the largest *R*_*i*_ = 100 µm indenter increases the indentation zone to well beyond the radiation damaged region (Fig. 4d). Thus in these measurements, the differences between the annealed and the irradiated samples are very small. This is not surprising since the indentation zone at yield in this test is dominated by layer D.

As described earlier, a major goal of our study was to develop and validate the nanoindentation data analysis methods to rigorously account for the crystal lattice orientation at the indentation site. We suggest that Fig. 3a and data shown in Fig. 5 capture this effect reliably and consistently, and could be used in studies on other samples of this material. The table in Fig. 5 summarizes the values of the indentation yield stress of the various layers in the radiation-affected region of the He-irradiated W sample; the values are compared between near-(100) and near-(111) oriented grains (all grains tested in this work had their surface normals within 6–14 degrees of the [100] and [111] directions). These two particular orientations were chosen since they exhibited the largest differences in the measured *Y*_*ind*_ values in prior studies on bcc metals^22,26,27^. As discussed earlier, the *Y*_*ind*_ values from *R*_*i*_ = 1 µm indenter are expected to be the best representation of the indentation yield strength of layer A. Similarly, the saturation stress for the *R*_*i*_ = 1 µm indenter is thought to represent the indentation yield strength of layer B. Both *R*_*i*_ = 5 and *R*_*i*_ = 10 µm indenters also show similar stress saturation levels. The beginning of the strain softening regions for both *R*_*i*_ = 5 and *R*_*i*_ = 10 µm indenters can be used as a surrogate for determining the depth of the ion-damaged region. Finally, the indentation yield strength of layer D is taken as the *Y*_*ind*_ values on annealed W using a *R*_*i*_ = 100 µm indenter that does not exhibit pop-ins.

Similar trends for stress-saturation have been reported in the literature for self-ion implanted tungsten, where the hardness values (HV) were found to saturate at around HV = 8.35–8.55 GPa^63^ to HV = 10.1–11.7 GPa^56^ for up to 33 dpa damage levels. Corroborative TEM images have also shown similar damage levels at these saturation dpa levels^56,63^. No effects of crystal orientation on hardness values were mentioned in these studies.

An important observation from the table in Fig. 5 are the differences in the measured *Y*_*ind*_ values of the two differently oriented grains, and their evolution with radiation damage in layers A, B and D. In the annealed condition (layer D), the average *Y*_*ind*_ values for the near (111) orientation are found to be ~29% higher than that of the near (100) orientation. This matches well with other prior studies on bcc metals that show a close to 30% difference between the hard (111) and soft (100) orientations^22,26,27^. However, this difference decreases dramatically with increasing amounts of radiation damage. In the transition damage layer A the two orientations differ only by 24%, while in layer B, where the damage is expected to be the greatest, the difference is only 12%. This observation points to significant differences in the damage experienced by grains of different orientations, i.e. a strong orientation effect of radiation induced mechanical changes at the grain scale. Obviously, surface energies for these two orientations are quite different. So there can be significant differences in the damage experienced by these grains, as seen in other reports^64,65^. Another possible explanation could be the extremely high defect density induced in the metal by irradiation^54,66,67^. Our preliminary (unreported) TEM investigations suggest that the He irradiation-induced defects in tungsten are both in the form of dislocation loops and He bubbles. Similar observations have also been reported in literature where the dislocations loops have been shown to coarsen and entangle with increased radiation dose^68^, and the He bubbles are distributed both uniformly and randomly^69–71^. It is interesting to note that the depth of He bubble region as determined by TEM is around 500 nm. This depth matches the transition between the hardening/saturation and softening regimes for the intermediate indenter sizes *R*_*i*_ = 5 and 10 µm (Fig. 4b and c). Also at these high levels of radiation damage (He bubble density ~8.5 × 10^23^ m^−3^ and size ~1.1 nm) all grains, irrespective of orientation, may saturate to a very large and similar level of strength^71^, leading to the trends seen in the table in Fig. 5. An exhaustive investigation of multiple grain orientations in tungsten, and their effects upon He implantation, is currently underway to explore these effects further.

In summary, the measurements shown in Figs 4 and 5 demonstrate the viability and tremendous potential of the spherical indentation stress-strain curves in investigating the changes in the mechanical response of nuclear materials with irradiation-induced surface damages. These methods are cost-effective in extracting huge amounts of reliable and consistently reproducible information from very small nanometer sample volumes. Compared to standard hardness measurements using sharper pyramidal indenters, which provide a variation of hardness and modulus with respect to indentation depth^16,55,56,63,72,73^, the indentation stress-strain analysis technique can provide us with the local loading and unloading elastic moduli, the local indentation yield strengths, and the post-yield strain hardening behavior and quantify these changes before and after radiation induced damage. Additionally, by simply varying the indenter size, this technique can be used to provide remarkable new insights into the mechanical response of the irradiated layers in these samples, and correlate those effects with the local material structure obtained from EBSD and/or TEM. As such, the ideas presented in this communication are applicable to all polycrystalline material systems (including metals and ceramics) with a modified surface layer. They can also be extended to a broad range of complex material systems where the local structure information is obtained by other materials characterization techniques (e.g., Raman-spectroscopy maps on bone^74^, back-scattered electron images).

## Materials and Methods

### Helium ion implantation

Helium ion implantation was performed on Danfysik Research Implanter at the Ion Beam Materials Laboratory (IBML) at LANL. The tungsten samples were irradiated to a relatively uniform box-like He concentration profile of 0.5 dpa at room temperature (see Fig. 1c). This was achieved by using four different He beam energies and fluences sequentially and additively: 200 keV at 2.0E16 ions/cm;^2^ 150 keV at 4.0E15 ions/cm;^2^ 100 keV at 8.0E15 ions/cm;^2^ and 50 keV at 7.2E15 ions/cm^2^. As a result, the He concentration in W was estimated to be ~0.92 atomic% between 150 nm and ~450 nm below the surface using SRIM (Stopping and Range of Ions in Matter) Monte Carlo code^75^.

The ion-radiation experiments were conducted on polycrystalline (grain size range 10–60 µm with average grain size 35 µm, see Fig. 2) samples of annealed (at 1500 °C for 3 days) and electro-polished (using a chilled sodium hydroxide solution at 8 V for 1 min^76,77^) tungsten. Note that an adequate surface preparation, such as electro-polishing or vibratory polishing that produces a smooth surface free of any additional strain due to the sample preparation techniques themselves^78^, is critical for obtaining reliable indentation stress-strain curves from spherical nanoindentation on metallic samples^45,46^. Annealing of tungsten is also known to increase the irradiation-induced hardening as compared to the as-received samples, owing to a lesser density of grain boundaries (which can act as sinks for interstitials and vacancies) in the annealed sample^55,79^. The choice of tungsten was motivated in part due to (i) its potential use as a fusion reactor first wall material, (ii) the isotropy of its elastic response at the single crystal level (iii) which has been reported to be unchanged after ion-irradiation^56^.

The He-implanted tungsten samples were investigated using TEM (FEI Tecnai F30). The He irradiation-induced defects in tungsten were found to be both in the form of dislocation loops and He bubbles (see Supplementary Information). In the uniformly damaged region for He implanted tungsten (between 150 nm and ~450 nm), the helium bubble density and size determined in the underfocus imaging condition^80^ were estimated to be ~8.5 × 10^23^ m^−3^ and ~1.1 nm, with the He bubbles ending at a depth of ~500 nm.

### Nanoindentation testing

Nanoindentation was carried out using the Agilent XP^®^ system maintained and operated by the Center for Integrated Nanotechnologies (CINT) at Los Alamos National Laboratory (LANL), Los Alamos, NM, USA, and equipped with the Continuous Stiffness Measurement (CSM) option. Four different spherical diamond tips with radii of *R*_*i*_ = 1, 5, 10, and 100 µm, respectively, were used in this study (see Fig. 2). As mentioned earlier the different radii of the indenters allow us to explore the influence of indentation zone length scales on the measurements. Multiple indentations (>20) were performed on each sample for each indenter size. The exact indent locations on the sample were verified using a combination of post-indentation EBSD (FEI XL30 Environmental Scanning Electron Microscope (ESEM)), SEM and/or optical micrography, as shown in Fig. 2b. Only indents located in the center of the grains, well away from any interfaces, were considered in the final analysis; indents that landed close to the grain boundaries were ignored.

### Data Analysis Protocols for calculating Indentation Stress-Strain (ISS) Curves

The ability to produce indentation stress-strain curves has generally been more successful with spherical indenters^34,81^, where the relatively smoother stress fields (compared to sharper indenters^82,83^) allow one to follow the evolution of the mechanical response in the material, from initial elasticity to the initiation of plasticity to post-yield behavior at finite plastic strains. The data analysis protocols used in Fig. 1 to convert the recorded load-displacement data to indentation stress-strain (ISS) curves can be summarized as a two-step procedure (see Refs^20,22^. for details). The first step in the analysis process is an accurate estimation of the point of effective initial contact in the given data set, i.e., a clear identification of a zero-point that makes the measurements in the initial elastic loading segment consistent with the predictions of Hertz’s theory^59,84,85^. As shown in Ref.^20^, the zero point can be conveniently determined using the following equation for the initial elastic segment in a frictionless, spherical indentation:

1 $$S=\frac{3P}{2{h}_{e}}=\frac{3(\tilde{P}-{P}^{\ast })}{2({\tilde{h}}_{e}-{h}^{\ast })}.$$

where $\tilde{P}$, ${\tilde{h}}_{e}$, and *S* are the measured load signal, the measured displacement signal, and the continuous stiffness measurement (CSM) signal in the initial elastic loading segment from the machine, respectively, and ${P}^{\ast }$ and ${h}^{\ast }$ denote the values of the load and displacement values at the point of effective initial contact. Rearrangement of Eq. (1) reveals that a plot of $\tilde{P}-\frac{2}{3}S{\tilde{h}}_{e}$ against *S* will produce a linear relationship whose slope is equal to $-\frac{2}{3}{h}^{\ast }$ and the *y*-intercept is equal to ${P}^{\ast }$. Therefore, a linear regression analysis can then be performed to identify the point of the effective initial contact (${P}^{\ast }$ and ${h}^{\ast }$) very accurately.

It is important to recognize that the effective zero-point defined here may not necessarily be the actual point of initial contact. The concept of an effective point of initial contact allows one to de-emphasize any artifacts created at the actual initial contact due to the unavoidable surface conditions (e.g., surface roughness, presence of an oxide layer) and imperfections in the indenter shape. It has to be interpreted as the point that brings the initial elastic loading segment to as close an agreement as possible with Hertz theory.

In the second step, the values of indentation stress and indentation strain can be calculated by recasting Hertz theory for frictionless, elastic, spherical indentation as

2 $$\begin{array}{ll}{\sigma }_{ind}={E}_{eff}{\varepsilon }_{ind}, & {\sigma }_{ind}=\frac{P}{\pi {a}^{2}},\quad {\varepsilon }_{ind}=\frac{4}{3\pi }\frac{{h}_{e}}{a}\approx \frac{{h}_{e}}{2.4a},\\ a=\frac{S}{2{E}_{eff}}, & \frac{1}{{E}_{eff}}=\frac{1-{\nu }_{s}^{2}}{{E}_{s}}+\frac{1-{\nu }_{i}^{2}}{{E}_{i}},\quad \frac{1}{{R}_{eff}}=\frac{1}{{R}_{i}}+\frac{1}{{R}_{s}}\end{array}$$

where ${\sigma }_{ind}$ and ${\varepsilon }_{ind}$ are the indentation stress and indentation strain, *a* is the radius of the contact boundary at the indentation load *P*, *h*_*e*_ is the elastic indentation depth, *S* (=*dP/dh*_*e*_) is the elastic stiffness described earlier, *R*_*eff*_ and *E*_*eff*_ are the effective radius and the effective stiffness of the indenter and the specimen system, $\nu$ and *E* are the Poisson’s ratio and the Young’s modulus, and the subscript*s s* and *i* refer to the specimen and the indenter, respectively.

A salient feature of the protocols described above is the use of CSM^23,86,87^ to obtain a reliable estimate of the radius of contact, *a*, at every point on the load-displacement curve (Eq. 2). The rigorous derivation of Eq. 2 directly from Hertz theory makes the estimates of contact radius from the measured CSM signals highly trustworthy.

## Electronic supplementary material

Supplementary Information
