# Supplementary material for: Probing nanoscale damage gradients in ion-irradiated metals using spherical nanoindentation
Source: Sci Rep. 2017 Sep 20;7:11918. doi: 10.1038/s41598-017-12071-6 (PMC5607315; doi:10.1038/s41598-017-12071-6)
Supplement: Supplementary file 1 — Supplementary Information [file 41598_2017_12071_MOESM1_ESM.pdf]

Probing nanoscale damage gradients in ion-irradiated metals using spherical nanoindentation

Siddhartha Pathak [a], Surya R. Kalidindi [b], Jordan S. Weaver [c], Yongqiang Wang [d], Russ Doerner [e], Nathan A. Mara [c,f]*

[a] Chemical and Materials Engineering, University of Nevada, Reno, NV, 89557, USA
[b] George W. Woodruff School of Mechanical Engineering, Georgia Institute of Technology, Atlanta, Georgia, GA 30332, USA
[c] Center for Integrated Nanotechnologies, Los Alamos National Laboratory, Los Alamos, NM, 87545, USA
[d] Materials Science and Technology Division, Los Alamos National Laboratory, Los Alamos, NM, 87545, USA
[e] Center for Energy Research, University of California at San Diego, La Jolla, CA. 92093, USA
[f] Institute for Materials Science, Los Alamos National Laboratory, Los Alamos , NM, 87545, USA

Supplementary Information:

Transmission Electron Microscopy (TEM) of He-implanted tungsten

The He-implanted tungsten samples were investigated using TEM (FEI Tecnai F30). The He irradiation-induced defects in tungsten were found to be both in the form of dislocation loops and He bubbles (**Fig. S1**). In the uniformly damaged region for He implanted tungsten (between 150 nm and ~450 nm), the helium bubble density and size

* Contact author: spathak@unr.edu, (775) 784-7098, 1664 N Virginia St, Mail Stop 0388, University of Nevada, Reno, Reno, NV 89557-0388 USA

determined in the underfocus imaging condition were estimated to be ~8.5x10^{23} m^{-3} and ~1.1 nm, with the He bubbles ending at a depth of ~500 nm.

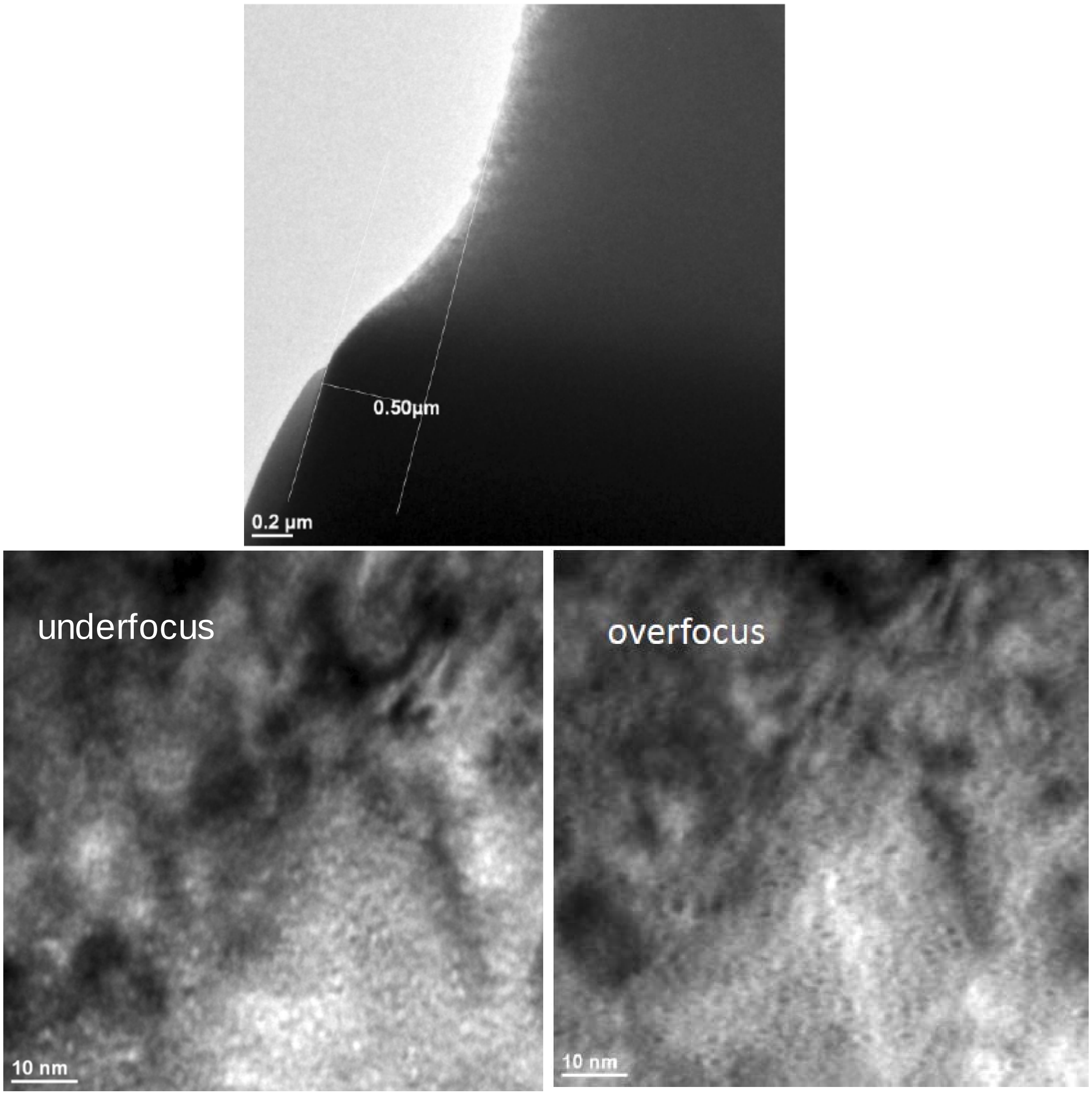

Figure S1. *TEM micrographs on He implanted tungsten. He bubbles end at a depth of ~ 500 nm*
